# Alpha-Glucosidase Inhibition, Antioxidant Activities, and Molecular Docking Study of Krom Luang Chumphon Khet Udomsak, a Thai Traditional Remedy

**DOI:** 10.1155/2024/1322310

**Published:** 2024-04-30

**Authors:** Thanchanok Limcharoen, Prapaporn Chaniad, Piriya Chonsut, Chuchard Punsawad, Thana Juckmeta, Atthaphon Konyanee, Ichwan Ridwan Rais, Surat Sangkaew

**Affiliations:** ^1^Department of Pharmaceutical Sciences and Technology Program, Faculty of Pharmaceutical Sciences, Chulalongkorn University, Bangkok 10330, Thailand; ^2^Department of Medical Sciences, School of Medicine, Walailak University, Nakhon Si Thammarat 80160, Thailand; ^3^Research Center in Tropical Pathobiology, Walailak University, Nakhon Si Thammarat 80160, Thailand; ^4^Department of Applied Thai Traditional Medicine, School of Medicine, Walailak University, Nakhon Si Thammarat 80160, Thailand; ^5^Department of Pharmaceutical Biology, Faculty of Pharmacy, Universitas Ahmad Dahlan, Yogyakarta 55164, Indonesia

## Abstract

Krom Luang Chumphon Khet Udomsak remedy (KKR) has traditionally been used as an alternative treatment, particularly for hyperglycemia; however, its therapeutic efficacy has not been scientifically validated. Thus, this study aims to investigate the potential inhibitory and antioxidant effects of *α*-glucosidase enzyme and characterize the chemical profile of KKR extracts using gas chromatography-mass spectrometry (GC-MS). The investigation highlights both KKR extracts as potent inhibitors of *α*-glucosidase, with the ethanolic extract of KKR (KKRE) displaying an IC_50_ value of 46.80 *µ*g/mL and a noncompetitive mode of action. The combination of ethanolic and aqueous extracts of KKR (KKRE and KKRA, respectively) with acarbose exhibited a synergistic effect against the *α*-glucosidase. The KKRE extract displayed strong scavenging effects in the DPPH assay (IC_50_ 156.3 *µ*g/mL) and contained significant total phenolic (172.82 mg GAE/g extract) and flavonoid (77.41 mg QE/g extract) contents. The major component of KKRE is palmitic acid (15.67%). Molecular docking revealed that the major compounds interacted with key amino acid residues (ASP215, GLU277, HIS351, ASP352, and ARG442), which are crucial for inhibiting *α*-glucosidase. Notably, campesterin had a more significant influence on *α*-glucosidase than acarbose, with low binding energy. These findings underscore the significance of KKR in traditional medicine and suggest that it is promising treatment for diabetes mellitus. Further studies using animal model will provide valuable insights for advancing this research.

## 1. Introduction

Diabetes mellitus (DM) is a global noncommunicable disease (NCD), affecting 422 million people in 2014, primarily in low- and middle-income nations, with a 13% increase in diabetes-related deaths between 2000 and 2019, causing 1.5 million deaths directly [1]. Projections estimate a 25% increase in DM cases by 2030 and a 51% increase by 2045 [2]. Diabetes mellitus, the most prevalent metabolic disorder, is characterized elevated blood glucose levels due to insulin imbalances [3]. Type 2 diabetes involves insulin resistance and insufficiency, leading to complications such as blindness, kidney failure, heart attack, stroke, and lower limb amputation. Prolonged hyperglycemia contributes to significant morbidity and mortality [4]. Chemical and synthetic medications have been developed to manage metabolic disorders [5].

*α*-Glucosidase inhibitors mitigate postmeal hyperglycemia in individuals with DM by impeding the inhibitory effect of *α*-glucosidase in the intestinal tract. This mechanism extends carbohydrate digestion, curtails glucose absorption, and delays glucose release into the bloodstream [6]. Notably, *α*-glucosidase inhibitors such as acarbose effectively lower postprandial blood glucose levels and are used in clinical practice [7]. Acarbose functions as a reversible inhibitor of intestinal *α*-glucosidases, which are enzymes pivotal for breaking down complex carbohydrates into absorbable monosaccharides. This mechanism leads to a reduced and delayed surge in blood glucose levels after meals, consequently lowering postprandial hyperglycemia. In addition, when combined with other antidiabetic therapies, such as sulfonylureas and insulin, acarbose has demonstrated additional efficacy in glycemic control. Commonly reported adverse reactions include abdominal pain, diarrhea, and flatulence, which typically decrease over time [8]. Despite the effectiveness of pharmaceutical drugs for blood glucose management, their extended use can lead to adverse effects [9]. Consequently, many people have turned to traditional herbal remedies because of their perceived safety and reduced side effects.

Krom Luang Chumphon Khet Udomsak remedy (KKR) is a traditional Thai herbal medicine used for centuries to manage various health conditions, including DM [10]. Thai herbs are rich in alkaloids and flavonoids and are valued for therapeutic properties [11]. Derived from herbal medicine scripture and Doctor Porn Krom Luang Chumphon Khet Udomsak's recipe, KKR aims to lower blood glucose levels. It comprised *Phyllanthus amarus* Schumach. and Thonn (PA), *Smilax corbularia* Kunth (SC), and *Smilax glabra* Roxb (SG), the evidence supporting KKR as a novel antidiabetic drug remains inconclusive. PA is known for its antidiabetic, antioxidant, anticancer, anti-inflammatory, and hepatoprotective properties [12]. Both SG and SC are rich in phenolic and flavonoid compounds, including catechin, astilbin, isoastilbin, taxifolin, and smiglasides, which are known for their antioxidant, anti-inflammatory, antibacterial, and anticancer properties [13]. While there is no scientific evidence supporting this remedy as an antidiabetic medicine, its potential in this area has not been fully explored. An initial study on its *α*-glucosidase inhibitory activity revealed promising effects, leading to a more detailed examination. Further analysis of the remedial extracts confirmed the strong *α*-glucosidase inhibitory effect of the KKR extract.

Thus, the primary objectives of this study are to evaluate the *α*-glucosidase enzyme inhibitory effect of KKR and its plant ingredient, elucidate the mechanisms of its principal botanical constituents, combination, and examine the interaction between these bioactive compounds and diabetic enzymes using molecular docking methodologies. The anticipated outcomes of this investigative study have the potential to facilitate the integration of this botanical resource into the development of food supplements and herbal medicines specifically designed to prevent hyperglycemia.

## 2. Materials and Methods

### 2.1. Materials

Thai Oil Co., Ltd., a Thai-based company, supplied all the necessary solvents for the extraction processes. The *α*-glucosidase enzyme (*Saccharomyces cerevisiae*), *p*-nitrophenol-*α*-glucopyranoside, acarbose, sodium carbonate, and dimethyl sulfoxide (DMSO) were procured from Sigma, Sigma-Aldrich (Germany). In addition, 1,1-diphenyl-2-picrylhydrazyl (DPPH) was acquired from Fluka, Sigma-Aldrich (USA), while phosphate-buffered saline (PBS) was sourced from Gibco® (Life Technologies, Paisley, Scotland).

### 2.2. Plant Material and Management

The botanical components within KKR were purchased from a legally registered Thai traditional herbal pharmacy situated in the Hatyai District, Songkhla Province, Thailand. These botanical constituents encompass *Phyllanthus amarus* Schumach. and Thonn, *Smilax corbularia* Kunth., and *Smilax glabra* Roxb. The authenticity of the plant materials was verified by a qualified and licensed traditional Thai pharmacist. As part of the reference collection process, samples of the remedies and each individual ingredient were gathered and subsequently preserved in the authors' herbarium, which was housed in the Department of Applied Thai Traditional Medicine, Walailak University, Nakhon Si Thammarat, Thailand (Table 1).

### 2.3. Preparation of Extraction

The extraction process was performed according to a meticulous protocol [14]. Plant specimens were washed and desiccated at 55°C for 24 h. After desiccation, the material was finely pulverized to ensure uniformity. KKR, which is a blend of plant components, weighs approximately 60 g. The preparation involved combining these components in equal proportions at a 1 : 1 : 1 ratio (20 g per plant component). One composite underwent ethanolic maceration, and the other was boiled in distilled water at 80°C for 6 hours. The extracts were rigorously filtered to remove impurities. The ethanolic extracts were subjected to rotary evaporation, and the aqueous extracts were freeze-dried. All extracts were stored at 4°C to preserve their bioactive constituents for subsequent bioassays.

### 2.4. *α*-Glucosidase Inhibitory Assay

The *α*-glucosidase inhibitory effect of KKR and its plant ingredient extracts was assessed using a colorimetric method [15]. All samples, including acarbose (a standard drug), were dissolved in a 20% DMSO solution. These solutions were dispensed into a 96-well plate, each containing 200 *µ*L, composed of 50 *µ*L PBS (pH 7.0), 50 *µ*L of 8 mg/mL sample solution (resulting in a final 2 mg/mL concentration), and 50 *µ*L *α*-glucosidase enzyme solution (at 1 U/mL). The plate was incubated at 37°C for 2 minutes, and then 50 *µ*L of 4 mM p-nitrophenyl-*α*-glucopyranoside solution was added. A microplate reader was used to measure the paranitrophenol content at 405 nm every 30 seconds for 10 minutes. The percent inhibition was calculated using the following equation:

(1) $$\begin{array}{c} \%\text{ Inhibition}=\left[\frac{\left(A_{405}^{\text{Control}}\;-A_{405}^{\text{Treatment}}\right)}{A_{405}^{\text{Control}}}\;\right]\times 100, \end{array}$$

where *A*_405_^Control^ is the absorbance at 405 nm in the control sample without the extract, and *A*_405_^Control^ is the absorbance at 405 nm after treatment with the extract.

### 2.5. Enzyme Kinetic Determination

The method for determining the mode of action and inhibition constant (Ki) against *α*-glucosidase inhibitory effect followed a precedent reference [16]. Both the Lineweaver–Burk equation and Dixon plot were employed, with a slight modification in the enzymatic reaction. Three concentrations of active crude extracts (25 to 500 *µ*g/mL) were chosen based on their observed activity. Six different substrate concentrations (0.15625–5 mM) were used. Constant amounts of *α*-glucosidase were incubated with increasing substrate concentrations (PNPG) at 37°C for 15 minutes, with or without samples (at concentrations equivalent to IC_50_). The necessary equations (2) and (3) were then applied to the analysis.

Line Weaver–Burk equation

(2) $$\begin{array}{c} \frac{1}{V}=\frac{\text{Km}}{V\max}\left(\frac{1}{S}\right)+\frac{1}{V\max}. \end{array}$$

Dixon equation

(3) $$\begin{array}{c} \frac{\text{Km}}{V\max}=\frac{\text{Km}}{V\max}\left(1+\frac{I}{\text{Ki}}\right). \end{array}$$

### 2.6. Combination Study

This study aimed to evaluate the effect of combining KKR extracts with acarbose, a standard drug, in inhibiting *α*-glucosidase. The IC_50_ values of acarbose and three concentrations of KKR (0.5–2IC_50_) were used to construct dose-response curves following the methodology of Chou [17]. The determination of combination index (CI) values was determined using CompuSyn software (version 1.0; https://www.combosyn.com/) accessed on December 27, 2023, which played a pivotal role. These CI values categorized the drug-drug combinations as synergistic (CI < 1), additive (CI = 1), or antagonistic (CI > 1) [17, 18]. The primary objective of this study was to provide insights into effective and safe strategies for managing diabetes, including the development of a novel Thai herbal remedy.

### 2.7. Antioxidant Activity (DPPH Radical Scavenging Assay)

The free radical scavenging capacities of the test samples were determined following an established protocol [19]. Each extract (100 *µ*L) and a positive control solution (0.1–100 *µ*g/mL) were combined in a 96-well plate. Then, 100 *µ*L of DPPH in methanol (6 × 10^−5^ M) was added and mixed thoroughly. The mixtures were kept in the dark for 30 minutes, and the absorbance was measured at 520 nm against a blank. Butylated hydroxytoluene (BHT) served as the standard. DPPH radical scavenging (RS) activity was determined using the following equation:

(4) $$\begin{array}{c} \%\text{ Inhibition}=\left[\frac{\left({\text{OD}}_{520}\text{ of Control }-\;{\text{OD}}_{520}\text{ of Sample}\right)}{{\text{OD}}_{520}\text{ of control}}\right]\times 100. \end{array}$$

### 2.8. Total Phenolic and Total Flavonoid Contents

Total phenolics and flavonoids in the crude extracts were evaluated Folin–Ciocalteu and aluminum chloride colorimetric methods [20, 21]. To estimate the total phenolic content, 100 *µ*L of samples were mixed with 500 *µ*L of 10% v/v Folin–Ciocalteu's reagent and 400 µL of 1 mM sodium bicarbonate. After incubation for 30 minutes, color intensity was measured at 765 nm. Gallic acid was used to generate a standard calibration curve. The total flavonoid content was quantified using the aluminum chloride colorimetric method. A mixture of 100 *µ*L 10% w/v aluminum chloride, 100 *µ*L 1 M potassium acetate, 1,500 *µ*L ethanol, and 500 µL samples was incubated for 30 minutes, and color intensity was measured at 415 nm. Quercetin served as the reference standard for calibration.

### 2.9. Gas Chromatography-Mass Spectrometric (GC-MS) Analysis

The analysis was conducted at the Scientific and Technological Research Equipment Centre of Chulalongkorn University in Bangkok, Thailand [22]. GC-MS analysis involved an Agilent Technologies 19091S-433 gas chromatograph (GC) coupled with an Agilent 5973 mass selective detector (MSD), controlled by Agilent Chemstation software. An HP-5MS capillary column (30 m length, 250 *µ*m inner diameter, 0.25 *µ*m film thickness) was used, and ultrapure helium served as the carrier gas at 0.7 mL/minute. The injector temperature was maintained at 300°C. The initial oven temperature was set at 50°C, increasing at 10°C/minute until reaching 310°C, and then held for 10 minutes. Injections of 1 *µ*L were in splitless mode (manual split ratio 10 : 1). The mass spectrometer was operated in the electron ionization mode at 70 eV, with an electron multiplier voltage set at 1859 V. Other parameters included an ion source temperature of 230°C, quadrupole temperature of 150°C, solvent delay of 4 minutes, and a scan range from 50 to 700 amu. Compounds in the test samples were identified by comparing their retention times (RT) and mass spectral data with those of standard compounds in the NIST library.

### 2.10. Computerized Molecular Docking

**Figure 1:**
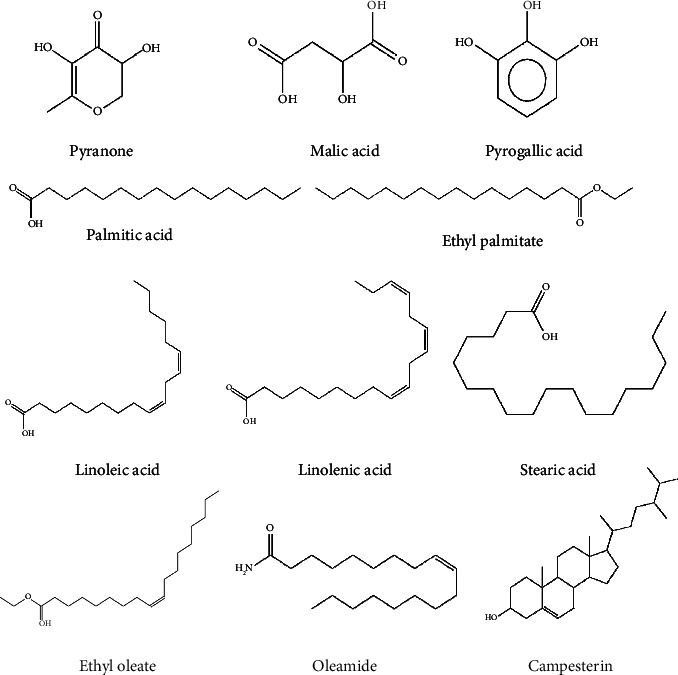
Structures of the compounds for molecular docking studies.

From GC-MS analysis, 11 compounds with peak areas exceeding 1% were identified for further exploration: pyranone, malic acid, pyrogallic acid, palmitic acid, ethyl palmitate, linoleic acid, linolenic acid, stearic acid, ethyl oleate, oleamide, and campesterin (Figure 1). The 3D crystal structure of *α*-glucosidase complexed with *α*-D-glucopyranose [23] (PDB ID: 3A4A, resolution 1.60 Å) was obtained from RCSB (https://www.rcsb.org/). The 3D structures of the identified compounds and acarbose were retrieved from PubChem (https://pubchem.ncbi.nlm.nih.gov/, accessed June 20, 2023). These structures were subjected to geometry optimization and energy minimization using GAFF [24] within Avogadro software version 1.2.0. [25]. Further preparation involved modifying the charges and torsion angles with AutoDockTools v.4.2.6. [26], saving the structures in the PDBQT format. The *α*-glucosidase structure was prepared using AutoDockTools, including removing cocrystallized ligands, eliminating water molecules, adding polar hydrogens, and assigning Kollman charges. Active amino acid regions, including ASP69, HIS112, ARG213, ASP215, GLU277, HIS351, ASP352, and ARG442 [23], were designated as active sites, and a grid box encompassing these regions was generated. The grid box specifications were set to 22 × 22 × 22 Å^3^ for *x*, *y*, and *z* coordinates, with a grid spacing of 1.000 Å and the following dimensions for the grid center (*x* = 21.348, *y* = −7.076, *z* = 23.432 Å). Molecular docking was performed using AutoDock Vina v.1.1.2 [27, 28], with an exhaustiveness value of 24 and default parameters. Compounds with the lowest binding energies and minimum RMSD were chosen as the best docking poses. The reliability of the docking process was confirmed by redocking *α*-D-glucopyranose into the active site, with an RMSD of 0.843 (RMSD <2.5 Å) [29]. Hydrogen and hydrophobic bond interactions were assessed using the PLIP online web server [30, 31]. The resulting protein-ligand complexes were visualized using the PyMOL Molecular Graphics System v.2.5.2. [32].

### 2.11. Statistical Analysis

In this study, we averaged the values from three separate experiments and expressed them as the mean ± standard deviation (SD). To analyze the data, we used a statistical method called two-way analysis of variance (ANOVA) along with Tukey's post hoc test specifically for total phenolic and flavonoid contents. For the remaining results, we applied a different statistical approach called one-way analysis of variance (ANOVA) using the GraphPad Prism software. The results were considered statistically significant if the *p* value was less than 0.05, which corresponds to a 95% confidence level.

## 3. Results

### 3.1. Percentage Yield

KKR and its plant ingredient extracts were subjected to a two-step process involving maceration in ethanol and boiling in distilled water. The dried weights of each extract and their respective percentage yields are listed in Table 2. The results reveal that, in each plant extract, aqueous extracts yielded higher percentages compared to the ethanolic extracts. Notably, the highest yield was obtained from the aqueous extract of *P. amarus* (9.91% w/w), whereas its ethanolic counterpart yielded only 8.20% w/w. For the KKR remedy itself, the aqueous extract exhibited a percentage yield of 6.11% w/w, while the ethanolic extract produced a percentage yield of 5.50% w/w.

### 3.2. *α*-Glucosidase Inhibitory Effect of the KKR and Its Plant Ingredients

**Figure 2:**
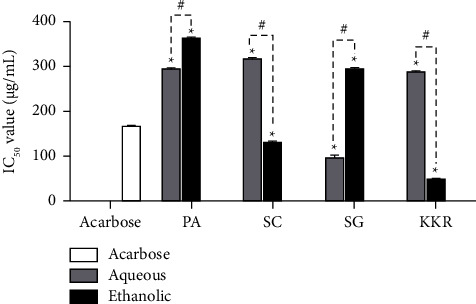
The *α*-glucosidase inhibitory activity of all extracts with standard drug acarbose. (PA: *P. amarus*, SC: *S. corbularia*, SG: *S. glabra*, and KKR: Krom Luang Chumphon Khet Udomsak remedy). (Statistical significance levels are indicated at ^*∗*^*p* < 0.0001 compared to acarbose (standard drug) and ^#^*p* < 0.0001 compared to the aqueous and ethanolic extracts).

The extraction of KKR and its plant ingredients were evaluated for their *α*-glucosidase inhibitory activity. Compared to the standard drug acarbose, with an IC_50_ value of 166.66 *µ*g/mL, the KKRE demonstrated the most potent inhibitory activity, with an IC_50_ value of 46.80 *µ*g/mL. Notably, the aqueous extract of *S. glabra* followed with a considerable inhibitory activity (IC_50_ 95.83 *µ*g/mL), along with the ethanolic extract of *S. glabra* (IC_50_ 131.44 *µ*g/mL). The KKRA and aqueous extract of *P. amarus* displayed strong activity with IC_50_ values of 288.49 and 292.55 *µ*g/mL, respectively. Furthermore, the ethanolic extract of *S. glabra*, the aqueous extract of *S. corbularia*, and the ethanolic extract of *P*. *amarus* exhibited moderate activity with IC_50_ values of 295.90, 316.14, and 362.33 *µ*g/mL, respectively (Figure 2).

### 3.3. Assessment of the Enzyme Kinetic Study of KKR

**Figure 3:**
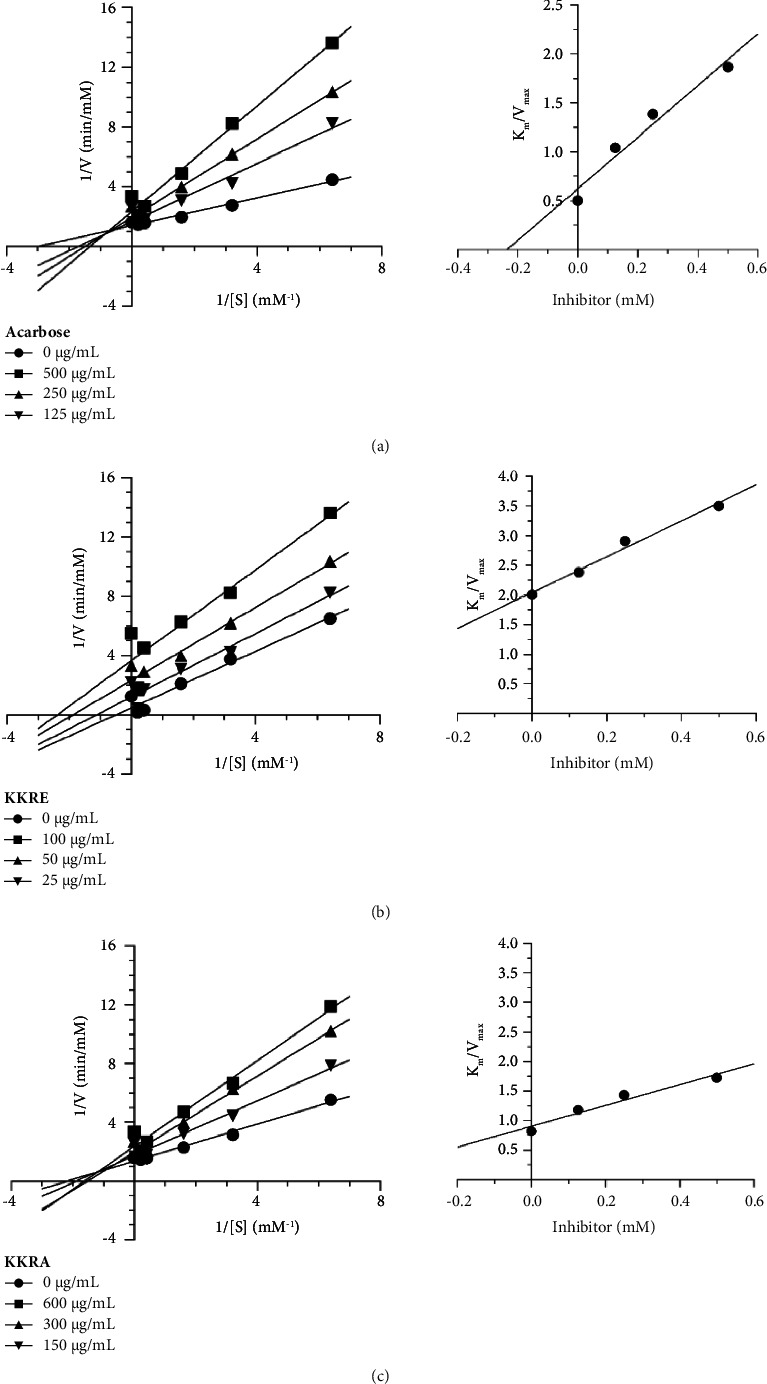
The mode of action of the standard drug, acarbose (a), and KKRE (b), and KKRA (c) extracts.

The enzyme kinetics study aimed to discern the type of inhibition exerted by the KKR extract, considering its traditional use as a remedy. Established methodologies, including the Lineweaver–Burk and Dixon equations, were used to elucidate the mode of action of the extracts. Analysis based on the Lineweaver–Burk equation revealed that KKRE exhibited noncompetitive inhibition with a Ki value of 0.672 mM. In contrast, the aqueous extract exhibited competitive inhibition, with a Ki value of 0.507 mM. Similarly, acarbose, a well-known inhibitor, demonstrated competitive inhibition, with a Ki value of 0.235 mM (Table 3 and Figure 3).

### 3.4. Combination Index Test of the KKR

**Figure 4:**
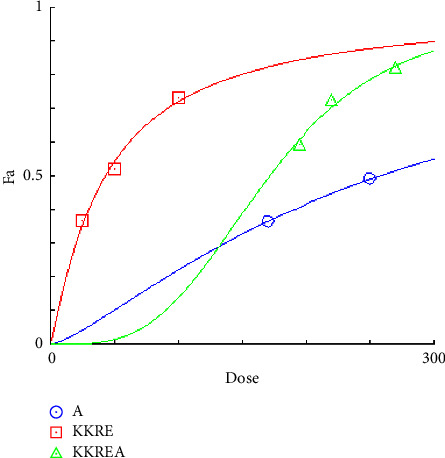
Dose-effect curve of constant combo acarbose-KKRE. (A: acarbose, KKRE: ethanolic extract of Krom Luang Chumphon Khet Udomsak remedy, KKREA: ethanolic extract of Krom Luang Chumphon Khet Udomsak remedy combined with acarbose, and Fa = the default effect).

**Figure 5:**
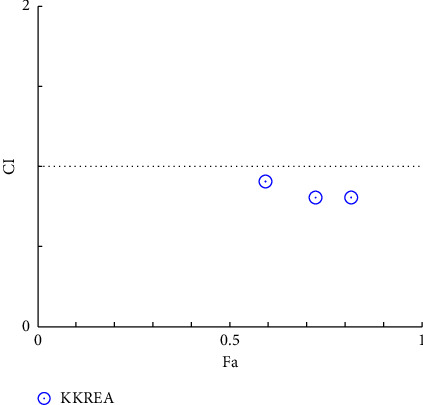
Combination index plot of constant combo acarbose-KKRE. (KKREA: ethanolic extract of Krom Luang Chumphon Khet Udomsak remedy combined with acarbose, CI: combination index, and Fa: the default effect).

**Figure 6:**
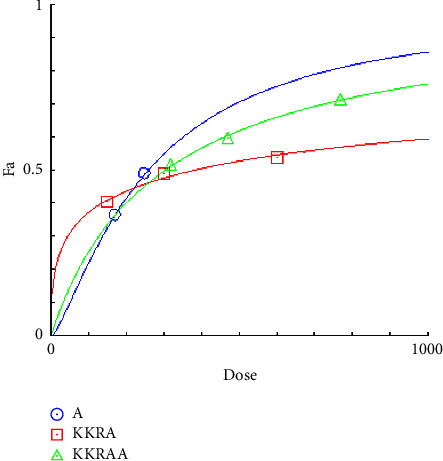
Dose-effect curve of constant combo acarbose-KKRA. (A: aqueous extract of Krom Luang Chumphon Khet Udomsak remedy, KKRAA: aqueous extract of Krom Luang Chumphon Khet Udomsak remedy combined with acarbose,and Fa = the default effect).

**Figure 7:**
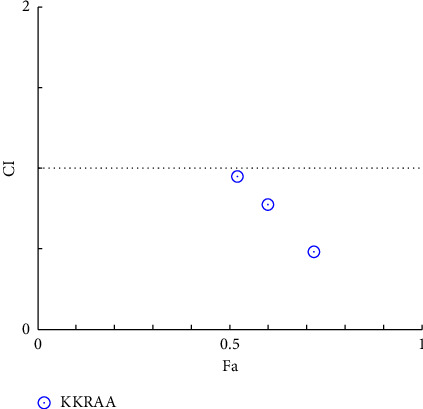
Combination index plot of constant combo acarbose-KKRA. (KKRAA: aqueous extract of Krom Luang Chumphon Khet Udomsak remedy combined with acarbose, CI: combination index, and Fa: the default effect).

Acarbose was utilized in the combination index (CI) analysis of the two extracts, KKRA and KKRE, in support of the traditional use of KKR. A fixed IC_50_ the amount of acarbose (170 µg/mL) was combined with varying amounts of each sample (which varied from IC_50_ 0.5–2). Tables 4 and 5 provide data on the CI test findings. As shown in Figure 4 (*y*-axis) and Figure 5 (*x*-axis), the combination of KKRE and acarbose resulted in a fraction affected (Fa) greater than 0.5. Similarly, as shown in Figure 6 (*y*-axis) and Figure 7 (*x*-axis), the combination of KKRA and acarbose generated Fa >0.5. These results indicated that >50% inhibitory activity was achieved by all combinations. For the KKRE, the CI values varied from 0.81 to 0.91. Moreover, it was also combined with acarbose. Moreover, CI values for KKRA were determined in the range of 0.48 to 0.95 when combined with acarbose. A synergistic effect was shown by KKR CI values of less than 1.

### 3.5. Antioxidant Activity as Determined by DPPH Radical Scavenging Assay

**Figure 8:**
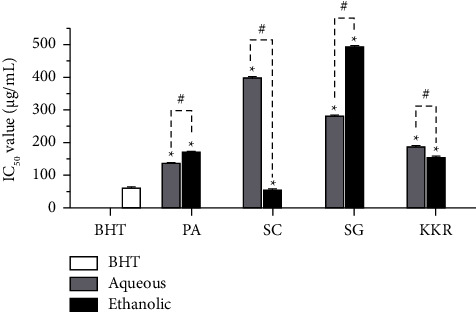
DPPH radical inhibitory effects of all extracts. (PA: *P. amarus*, SC: *S. corbularia*, SG: *S. glabra*, and KKR: Krom Luang Chumphon Khet Udomsak remedy). (Statistical significance levels are indicated at ^*∗*^*p* < 0.0001 compared to acarbose (standard drug) and ^#^*p* < 0.0001 compared to the aqueous and ethanolic extracts).

The antioxidant activities of all the extracts varied (Figure 8). The IC_50_ values, representing the DPPH radical scavenging activity, ranged from 53.52 to 495.19 *µ*g/mL. Among the extracts, the ethanolic extract of *S. corbularia* exhibited the highest scavenging effect, with a value of 53.52 *µ*g/mL, followed by the aqueous extract of *P. amarus* (138.57 *µ*g/mL). Decreasing scavenging effects were observed for the extract of KKRE (156.3 *µ*g/mL), ethanolic extract of *P. amarus*, extract of KKRA, aqueous extract of *S. glabra*, aqueous extract of *S. corbularia*, and ethanolic extract of *S. glabra*, respectively. The positive control, BHT, inhibited DPPH radical scavenging with an IC_50_ value of 62.24 *µ*g/mL.

### 3.6. Total Phenolic and Total Flavonoid Contents

**Figure 9:**
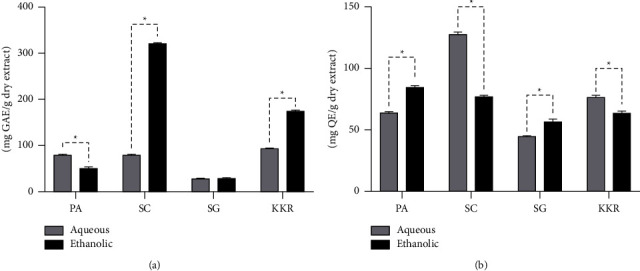
Total phenolic (a) and total flavonoid (b) contents of KKR and its components (PA: *P. amarus*, SC: *S. corbularia*, and SG: *S. glabra*, KKR: Krom Luang Chumphon Khet Udomsak remedy) (Statistical significance levels are indicated at ^*∗*^*p* < 0.0001 when comparing the ethanolic and aqueous extracts).

The quantification of total phenolic and flavonoid contents in the extracts of KKR and its individual components is presented in Figure 9. For the total phenolic content, the observed values varied within a range of 30.72 mg GAE/g to 319.50 mg GAE/g. The highest total phenolic content was recorded for the ethanolic extract of *S. corbularia*, measuring 319.50 mg GAE/g, followed by KKRE (172.82 mg GAE/g). In contrast, the lowest value, (30.72 mg GAE/g) was observed in the ethanolic extract of *S. glaba*. This figure also shows the flavonoid content. The ethanolic extract of *S. corbularia* exhibited the highest value (128.40 mg QE/g), followed by the aqueous extract of *P. amarus* at 85.49 mg QE/g. The total flavonoid content of KKRE was 77.41 mg QE/g.

### 3.7. Gas Chromatography-Mass Spectrometric (GC-MS) Analysis

**Figure 10:**
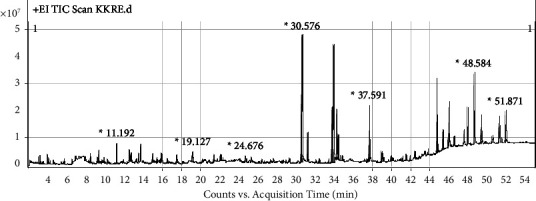
GC-MS chromatogram of the KKRE.

The extract of KKRE demonstrated the most significant impact on *α*-glucosidase activity. Subsequently, gas chromatography-mass spectrometry (GC-MS) was conducted to determine its chemical composition. The analysis revealed 86 compounds, of which 32 exhibited a matching score of >80%.  Figure 10 shows the chromatogram of the compounds identified in the KKRE extract. The analysis identified 32 compounds, which are listed in Table 6. The most abundant compound detected was *n*-hexadecanoic acid, also known as palmitic acid, with a retention time of 30.576 minute, constituting 15.67% of the total compound. Linolenic acid (14.06%), linoleic acid (6.75%), oleamide (3.71%), stearic acid (3.62%), oleic acid, and ethyl ester (1.75%) were identified as the significant constituents.

### 3.8. Molecular Docking

**Figure 11:**
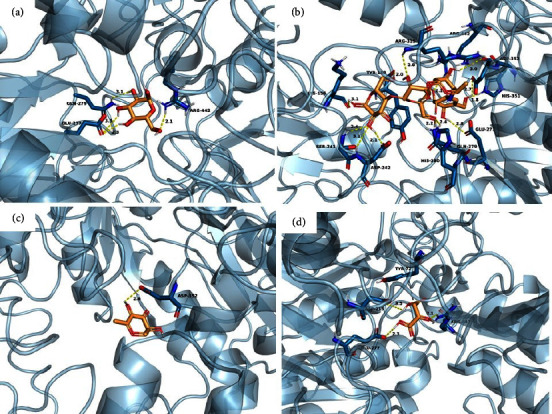
Predicted binding modes, H-bond and hydrophobic interactions of *α*-D-glucopyranose (a), acarbose (b), pyranone (c), and malic acid (d) with *α*-glucosidase. The backbone of the *α*-glucosidase enzyme is presented in a blue-ribbon model, the yellow line dot represents the hydrogen bond, and the gray dot represents the hydrophobic interaction.

**Figure 12:**
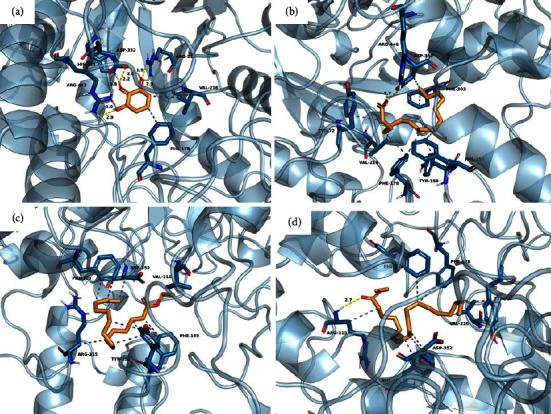
Predicted binding modes, H-bond and hydrophobic interactions of pyrogallic acid (a), palmitic acid (b), ethyl palmitate (c), and ethyl oleate (d) with *α*-glucosidase. The backbone of the *α*-glucosidase enzyme is presented in a blue-ribbon model, the yellow line dot represents the hydrogen bond, and the gray dot represents the hydrophobic interaction.

**Figure 13:**
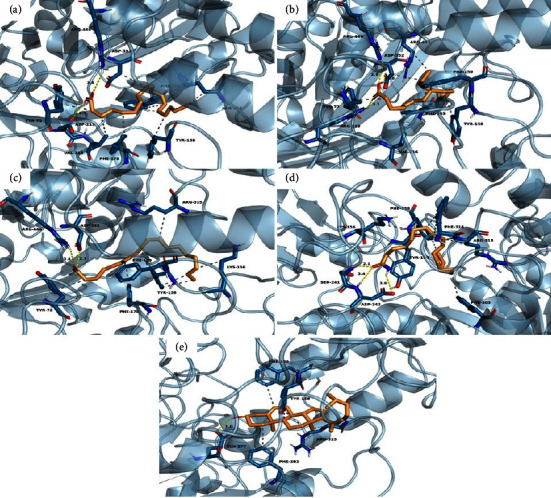
Predicted binding modes, H-bond and hydrophobic interactions of linoleic acid (a), linolenic acid (b), stearic acid (c), oleamide (d), and campesterin (e) with *α*-glucosidase. The backbone of the *α*-glucosidase enzyme is presented in a blue-ribbon model, the yellow line dot represents the hydrogen bond, and the gray dot represents the hydrophobic interaction.

Eleven compounds present in KKRE that exhibited peak areas greater than 1% were selected for molecular docking.  Table 7 provides information on the binding energy and amino acid residues of the *α*-glucosidase enzyme that interacts with each compound, including details of the hydrogen bonds and hydrophobic interactions. To identify the interacting amino acid residues and predict the binding modes of the compounds with *α*-glucosidase, 2D interaction diagrams were generated, as shown in Figures 11–13. Among the eleven compounds, campesterin demonstrated the strongest binding affinity to the *α*-glucosidase enzyme, with a low binding energy of −8.9 kcal/mol, representing the highest observed affinity to an enzyme. Its affinity surpasses that of acarbose, the standard drug, which exhibits a binding energy of −8.4 kcal/mol. Campesterin formed a hydrogen bond with GLU277 and exhibited hydrophobic interactions with TYR158, PHE159, PHE303, and ARG315 (Figure 13(e)), which contributed to its stabilization. Linolenic acid displayed a binding affinity to the *α*-glucosidase enzyme with a binding energy of −6.8 kcal/mol. It strongly interacts with ARG213, ASP352, and ARG446 (Figure 13(b)). This compound engages in eight hydrophobic interactions with residues TYR72, TYR158, PHE159, VAL216, PHE303, ASP352, and ARG442. Pyrogallic acid exhibited notable binding affinity to the *α*-glucosidase enzyme, forming seven hydrogen bonds with ARG213, HIS351, ASP352, and ARG442, and possessing a binding energy of −5.9 kcal/mol (Figure 12(a)). In addition, it engages in two hydrophobic interactions with residues PHE178 and VAL216. However, it is worth noting that these compounds interacted with fewer amino acids compared to acarbose, which formed thirteen hydrogen bonds with LYS156, TYR158, SER241, ASP242, GLU277, GLN279, HIS280, ARG315, HIS351, ASP352, and ARG442, resulting in a binding energy of −8.4 kcal/mol (Figure 11(b)).

## 4. Discussion

Delaying carbohydrate digestion and reducing glucose absorption through the inhibition of *α*-glucosidase represents a therapeutic avenue for managing type 2 diabetes (T2DM). Numerous studies have documented the effect of herbal medicine on the suppression of *α*-glucosidases [33, 34]. Krom Luang Chumphon Khet Udomsak remedy (KKR) has been used in Thai traditional medicine for centuries to treat DM [10]. The present study was undertaken to evaluate the potential anti-*α*-glucosidase and antioxidant activities of KKR and its plant ingredients, with a series of experiments conducted to substantiate their biological effects. From our results, both KKR remedies demonstrated a remarkably *α*-glucosidase inhibitory activity. These results suggest that the KKRE extract exhibited the highest inhibition of *α*-glucosidase activity through noncompetitive mechanisms, surpassing other extracts and demonstrating an IC_50_ lower than that of the standard drug acarbose. The presence of palmitic acid, identified as a major component of KKRE through GC-MS analysis, suggests its potential to inhibit *α*-glucosidase, as previously reported [35]. Importantly, the Ki value of KKRE associated with this mechanism was significantly higher than that of acarbose, suggesting a relatively low binding affinity of this extract for the enzyme [36].

Moreover, our findings underscore the efficacy of ethanol as the optimal solvent for extracting secondary metabolites from KKR and its plant constituents, particularly evident in our observations of anti-*α*-glucosidase and antioxidant activities. This preference for ethanol arises from its intrinsic polarity, which allows the selective isolation of low molecular weight phenolic and flavonoid compounds [37]. These compounds are prevalent in plants and are associated with antidiabetic [38] and antioxidant [39] properties in biological systems.

Furthermore, our assessment of *α*-glucosidase inhibition across all extracts revealed that KKRA exhibited potent inhibition of the *α*-glucosidase enzyme, while KKRE demonstrated significantly higher *α*-glucosidase inhibitory activity compared to other extracts. Similarly, in terms of antioxidant activity, the ethanolic extract of *S*. *corbularia* displayed the highest efficacy. Palmitic acid, a fatty acid soluble in organic solvents [40], has been identified as a significant contributor to *α*-glucosidase inhibition [35]. This explains the superior results observed with the ethanolic extract of KKR and other plant components compared with the aqueous extract. In a previous study investigating herbal components, it was observed that the powdered ash obtained from *P*. *amarus* displayed inhibitory activity against *α*-glucosidase, with an IC_50_ value of 982.13 ± 162.69 *µ*g/mL [41]. In addition, the ethyl acetate rhizome extract of *S. glabra* demonstrated inhibitory activity against *α*-glucosidase, with an IC_50_ value of 5.5 *µ*g/mL [42]. Furthermore, the ethanolic rhizome extract of *S. corbularia*, at a concentration of 25 *µ*g/mL, also displayed inhibitory activity against *α*-glucosidase at approximately 50% [43]. Consequently, based on the results of the *α*-glucosidase inhibitory assay, it was determined that the KKR had potent inhibitory effects on the *α*-glucosidase enzyme.

This observation aligns with previous research suggesting that phenolic and flavonoid compounds can act as inhibitors of *α*-glucosidase, contributing to the regulation of hyperglycemia [44] and also can act as inhibitors of antioxidants activity [45].

Since, oxidative stress has been demonstrated to participate in the progression of diabetes which plays an important role during diabetes, including impairment of insulin action and elevation of the complication incidence [46]. Oxidative stress involves the transfer of hydrogen or electrons from stable molecules to free radicals, which then convert them into stable molecules [47]. Therefore, we assessed the DPPH free radical scavenging ability of KKR and its plant ingredients. According to our findings, the ethanolic extract of *S. corbularia* demonstrated the highest inhibition of DPPH activity compared to the other extracts, and also exhibited the lowest IC_50_ compared to the positive control, BHT. The observed DPPH radical scavenging capacity of the ethanolic extract derived from *S. corbularia* rhizome can be attributed to the presence of polyphenolic compounds within the plant material as reported previously [48]. As per a prior investigation, the ethanolic extract derived from the rhizome of *S. corbularia* demonstrated significant potency in inhibiting DPPH, with an IC_50_ value of 9.24 ± 1.71 at concentrations ranging from 0.15 to 75 *µ*g/mL [43]. In our study, we observed that the ethanolic extract from *S. corbularia*, tested at concentrations ranging from 0.1 to 100 µg/mL, also exhibited notable potency in inhibiting DPPH, with an IC_50_ value of 53.52 ± 0.59. Plants are abundant in polyphenols and flavonoids, exhibit strong antioxidant activity, and have diverse defense and disease-fighting properties [49]. Phenolics and flavonoids present in medicinal plants and foods are essential components that contribute to a range of antidiabetic activities [50]. Elevated levels of total phenolic content (TPC) and total flavonoid content (TFC) serve as indicators of the potential therapeutic activities inherent in plant extracts [51]. In a previous investigation on the TPC and TFC of the herbal components, it was determined that the ethanolic extract of *S. corbularia* contained quantifiable amounts of phenolic and flavonoid compounds, with concentrations measuring 388.22 ± 1.92 mg GAE/g extract and 33.72 ± 1.18 mg QE/g extract, respectively. Similarly, the ethanolic extract of *S. glabra* was found to contain detectable levels of phenolic and flavonoid compounds, with concentrations of 30.83 ± 1.18 mg GAE/g extract and 21.12 ± 0.54 mg QE/g extract, respectively [43]. These findings corroborate our observations that the ethanolic extract of *S. corbularia* exhibited higher TPC and TFC levels than those of *S. glabra*.

GC-MS of the KKRE extract, which had the highest potential to inhibit *α*-glucosidase enzyme, revealed 86 compounds, among which 32 exhibited a matching score of more than 80%. Eleven compounds were selected for a better understanding of the compounds in KKRE, and molecular docking was performed. These compounds included pyranone, malic acid, pyrogallic acid, palmitic acid, ethyl palmitate, linoleic acid, linolenic acid, stearic acid, ethyl oleate, oleamide, and campesterin. Consequently, it can be inferred that KKRE induces a reduction in glucose levels in the presence of these compounds. The main chemical constituents identified in KKRE comprised palmitic acid, as reported in prior studies investigating the ethanolic leaf extract of *P. amarus* [52] and the methanolic extract of the *Smilax China* plant within the Smilax genus [53]. Furthermore, margarinic acid was documented in the methanolic extract of *Smilax zeylanica* [54], while malic acid and oleic acid ethyl ester were also detected in the rhizome of the ethanolic *Smilax domingensis* extract [55]. The chemical constituents of KKRE exhibited *α*-glucosidase inhibitory activity; for instance, palmitic acid demonstrated potential inhibitory effects on the *α*-glucosidase enzyme [35], while malic acid also showed similar inhibitory potential [56]. To explore the interactions of the identified compounds with the *α*-glucosidase enzyme, we employed molecular docking, an integral part of *in silico* drug development to predict small molecule-protein interactions at the atomic level [57]. Docking results revealed that campesterin exhibited the strongest binding affinity to the *α*-glucosidase enzyme, with a low binding energy of −8.9 kcal/mol. It forms a hydrogen bond with GLU277 and engages in hydrophobic interactions with residues TYR158, PHE159, PHE303, and ARG315. This finding aligns with previous reports on the antidiabetic, cholesterol-lowering, anticarcinogenic [58], antioxidant, antibacterial, immunomodulatory, and anti-inflammatory activities [59]. In addition, linoleic acid has been identified as a multi-target inhibitor associated with insulin resistance [60].

There has been a hypothesis that two inhibitors, each employing different modes of inhibition, might synergistically contribute to the inhibition of *α*-glucosidase [61, 62]. To test this hypothesis, we performed a combined assay. The addition of KKR to the enzymatic reactions with acarbose revealed a synergistic effect, as evidenced by the CI value <1. Our findings revealed, for the first time, the potential of KKR extracts as a herbal medicinal therapy for diabetes. In addition, this information could offer healthcare professionals valuable insights into the potential use of KKR in combination with the antidiabetic agent acarbose to enhance the control of blood glucose levels in diabetic patients. This knowledge may further contribute to our understanding of the potential synergies between KKR and other bioactive substances and medications.

## 5. Conclusions

This study represents the first investigation of the biological activities of KKR, a Traditional Thai herbal remedy with a century-old history of managing various health conditions. Our findings indicate that KKRA exerts potent inhibitory effects on the *α*-glucosidase via a competitive mechanism. In addition, KKRE exhibited the highest potency in inhibiting the *α*-glucosidase enzyme through a noncompetitive mechanism compared with the standard drug acarbose. Notably, the combination of KKR extract and acarbose had a remarkable synergistic effect on *α*-glucosidase inhibition. KKR and its plant ingredients exhibit potent antioxidant activities. Furthermore, a molecular docking study identifies campesterin, a constituent compound from KKRE, as having the most significant impact on *α*-glucosidase inhibition. These outcomes provide substantial evidence of the importance of KKR in traditional medicine and underscore its potential significance in contemporary therapeutic contexts.

## Figures and Tables

**Table 1:** List of plant materials used in the study.

No	Plant species	Family	Plant part	Voucher number
1	*Phyllanthus amarus* Schum and Thonn	Euphorbiaceae	Whole plant	SMD 290 055 004
2	*Smilax corbularia* Kunth	Smilacaceae	Rhizome	SMD 261 002007
3	*Smilax glabra* Roxb	Smilacaceae	Rhizome	SMD 261 002011

**Table 2:** Extraction yields of ethanolic and aqueous extracts of the medicinal plants in KKR.

Plant species	Extraction yield (% w/w)	
	Ethanolic extract	Aqueous extract
*P*. *amarus*	8.20	9.91
*S*. *corbularia*	4.98	5.46
*S. glabra*	4.11	4.71
KKR	5.50	6.11

**Table 3:** The presented data showcases the kinetic parameters of *α*-glucosidase when exposed to the extract of KKRE and acarbose (standard drug).

Inhibitors	*α*-Glucosidase	
	Ki (mM)	Mode
Acarbose	0.235	Competitive
KKRE	0.672	Noncompetitive
KKRA	0.507	Competitive

**Table 4:** Combination index (CI) of standard drug (acarbose) and KKRE.

Name	Sample; KKRE				
Standard; acarbose	Concentration (*µ*g/mL)		25	50	100
		% Inhibition (CI)^*∗*^	36.78	52.19	73.14
	170	36.55	59.51 (0.91)^*∗*^	72.55 (0.80)^*∗*^	82.09 (0.81)^*∗*^

^*∗*^CI = Combination index.

**Table 5:** Combination index (CI) of standard drug (acarbose) and KKRA.

Name	Sample; KKRA				
Standard; acarbose	Concentration (*µ*g/mL)		150	300	600
		% Inhibition (CI)^*∗*^	40.45	49.06	54.08
	170	36.55	51.98 (0.95)^*∗*^	59.88 (0.78)^*∗*^	71.64 (0.48)^*∗*^

^*∗*^CI = Combination index.

**Table 6:** Compounds identified in the KKRE by GC-MS.

Peak	RT (min)	Name of the compounds	Molecular formula	Molecular weight	Peak area (%)
1	3.199	Lactonitrile	C_3_H_5_NO	71	0.32
2	3.951	Furfural	C_5_H_4_O_2_	96	0.36
3	4.095	Dimethyl sulfoxide	C_2_H_6_OS	78	0.36
4	4.276	Furfuryl alcohol	C_5_H_6_O_2_	98	0.12
5	5.707	2,2-Diethoxyethanol	C_6_H_14_O_3_	134	0.22
6	6.527	2-Furaldehyde,5-methyl-	C_6_H_6_O_2_	110	0.19
7	8.438	1-Amino-2,6-dimethylpiperidine	C_7_H_16_N_2_	128	0.73
8	10.221	6-Deoxyhexopyranose	C_6_H_12_O_5_	164	0.45
9	11.192	Pyranone	C_6_H_8_O_4_	144	1.19
10	12.514	Ethyl 3-hydroxy-2,2-dimethylbutanoate	C_8_H_16_O_3_	160	0.61
11	13.696	2-Coumaranone	C_8_H_6_O_2_	134	0.99
12	14.984	Malic acid	C_4_H_6_O_5_	134	1.04
13	15.403	Salicylic acid	C_7_H_6_O_3_	138	0.26
14	17.454	Pyrogalic acid	C_6_H_6_O_3_	126	1.13
15	20.865	2,4-d-t-Butylphenol	C_14_H_22_O	206	0.22
16	21.700	Ochracin	C_10_H_10_O_3_	178	0.17
17	22.051	Vanillic acid	C_8_H_8_O_4_	168	0.82
18	27.577	3,4,5-Trimetthoxybenzyl alcohol	C_10_H_14_O_4_	198	0.33
19	28.476	Pentadecylic acid	C_15_H_30_O_2_	242	0.10
20	28.597	Cinnamic acid	C_10_H_10_O_4_	194	0.18
21	28.891	Spiro [tricyclo[4.4.0.0(5,9)] decane-10,2′-oxirane], 1-methyl-4-isopropyl-7,8-dihydroxy-, (8S)-	C_15_H_24_O_3_	252	0.17
22	30.576	Palmitic acid	C_16_H_32_O_2_	256	15.67
23	31.150	Ethyl palmitate	C_18_H_36_O_2_	284	1.62
24	32.355	Margarinic acid	C_17_H_34_O_2_	270	0.29
25	33.341	Phytol	C_20_H_40_O	296	0.30
26	33.742	Linoleic acid	C_18_H_32_O	280	6.75
27	33.862	Linolenic acid	C_18_H_30_O	278	14.06
28	34.218	Stearic acid	C_18_H_36_O_2_	284	3.62
29	34.338	Oleic acid, ethyl ester	C_20_H_38_O_2_	310	1.75
30	34.788	Ethyl stearate	C_20_H_40_O_2_	312	0.29
31	37.591	Oleamide	C_18_H_35_NO	281	3.71
32	47.500	Campesterin	C_28_H_48_O	400	1.08

RT: Retention time.

**Table 7:** The binding energy and amino acid residues of the *α*-glucosidase enzyme.

Compound	Binding energy (kcal/mol)	H-bond interaction		Hydrophobic interaction	
		Number of interaction	Amino acid residues	Number of interaction	Amino acid residues
*α*-D-glucopyranose (co-crystallized ligand)	−5.7	4	GLU277^a^, GLN279, ARG442	—	—
Acarbose (standard drug)	−8.4	13	LYS156, TYR158, SER241, ASP242^a^, GLU277, GLN279, HIS280, ARG315, HIS351, ASP352^a^, ARG442	—	—
Pyranone	−5.3	1	ASP352	—	—
Malic acid	−5.4	3	ASP215, GLU277, ARG442	1	TYR72
Pyrogallic acid	−5.9	7	ARG213^a^, HIS351, ASP352^a^, ARG442^a^	2	PHE178, VAL216
Palmitic acid	−6.1	1	ARG446	8	TYR72, TYR158, PHE159, PHE178^a^, VAL216, PHE303, ASP352
Ethyl palmitate	−5.3	—	—	9	TYR158^b^, PHE159, VAL216, PHE303^a^, ARG315, ASP352
Linoleic acid	−6.5	2	ASP215, ARG446	8	TYR72, TYR158, PHE178, VAL216, PHE303^a^, ARG315, ASP352
Linolenic acid	−6.8	3	ARG213, ASP352, ARG446	8	TYR72, TYR158, PHE159, VAL216, PHE303^a^, ASP352, ARG442
Stearic acid	−6.5	2	ASP352, ARG446	8	TYR72, LYS156, TYR158^b^, PHE159, PHE178, ARG315
Ethyl oleate	−6.0	1	ARG315	8	TYR72, PHE159, PHE178, VAL216, PHE303^a^, ARG315, ASP352
Oleamide	−6.1	3	SER241, ASP242^a^	7	LYS156, TYR158, PHE159, PHE303, PHE314^a^, ARG315
Campesterin	−8.9	1	GLU277	5	TYR158^a^, PHE159, PHE303, ARG315

^a^Two interaction with amino acid residues. ^b^Three interaction with amino acid residues.

## Data Availability

The data used to support the findings of this study are included within the article.

## Abbreviations

DM: Diabetes mellitus
NCDs: Noncommunicable diseases
WHO: World Health Organization
KKR: Krom Luang Chumphon Khet Udomsak remedy
KKRE: Ethanolic extract of Krom Luang Chumphon Khet Udomsak remedy
KKRA: Aqueous extract of Krom Luang Chumphon Khet Udomsak remedy
PA: *Phyllanthus amarus* Schumach. and Thonn
SC: *Smilax corbularia* Kunth
SG: *Smilax glabra* Roxb
DMSO: Dimethyl sulfoxide
DPPH: 1,1-diphenyl-2-picrylhydrazyl
PBS: Phosphate-buffered saline
Ki: Inhibition constant
PNG: *Para*-nitrophenyl-*α*-glucopyranoside
BHT: Butylated hydroxytoluene
RS: Radical scavenging
IC_50_: Half maximal inhibitory concentration
GC-MS: Gas chromatography-mass spectrometry
MSD: Mass selective detector
NIST: National Institute of Standards and Technology
RIIE: Research and Innovation Institute Excellence.
